# Focal segmental glomerulosclerosis: molecular genetics and targeted therapies

**DOI:** 10.1186/s12882-015-0090-9

**Published:** 2015-07-09

**Authors:** Ying Maggie Chen, Helen Liapis

**Affiliations:** 1 grid.4367.60000000123557002Renal Division, Washington University School of Medicine, 660 S. Euclid Ave., St. Louis, MO 63110 USA; 2 Nephropath, Little Rock Arkansas; 3 grid.4367.60000000123557002Pathology & Immunology, Washington University School of Medicine, St. Louis, MO USA

**Keywords:** Focal segmental glomerulosclerosis, Podocyte gene mutation, Proteinuria, Next-generation sequencing

## Abstract

Recent advances show that human focal segmental glomerulosclerosis (FSGS) is a primary podocytopathy caused by podocyte-specific gene mutations including *NPHS1*, *NPHS2*, *WT-1*, *LAMB2*, *CD2AP*, *TRPC6*, *ACTN4* and *INF2*. This review focuses on genes discovered in the investigation of complex FSGS pathomechanisms that may have implications for the current FSGS classification scheme. It also recounts recent recommendations for clinical management of FSGS based on translational studies and clinical trials. The advent of next-generation sequencing promises to provide nephrologists with rapid and novel approaches for the diagnosis and treatment of FSGS. A stratified and targeted approach based on the underlying molecular defects is evolving.

## **Review**

Focal segmental glomerulosclerosis (FSGS) was first recognized in the 20^th^ century as a histopathological pattern of glomerular injury associated with nephrotic syndrome (NS) [1]. It is a lesion rather than a disease with morphologic variations including tip, perihilar, cellular, collapsing, and not otherwise specified (NOS) features [2]. The most common manifestation of FSGS is proteinuria, which may range from subnephrotic to nephrotic levels [3]. NS, characterized by heavy proteinuria, hypoalbuminemia and hyperlipidemia, often leads to progressive loss of kidney function, accounting for ~15 % of end-stage renal disease (ESRD). The cost to health care exceeds $3 billion in the U.S. annually [4, 5]. FSGS accounts for 7-20 % of idiopathic NS in children and 40 % in adults and is the most common glomerular disease leading to ESRD in African Americans (AAs) [6, 7]. Since the original description was based only on morphology, numerous studies were conducted to understand the pathogenesis of FSGS. In this review we focus on recent molecular insights into FSGS pathogenesis including results from our studies and discuss the effects on current treatment of patients with FSGS.

### Structural and functional podocyte defects in FSGS

Diverse clinicopathologic etiologies lead to FSGS (Table 1). Primary (idiopathic) FSGS is due to defects inherent in the podocyte structure or function. FSGS secondary to genetic causes, circulating permeability factor(s), hemodynamic adaptations causing glomerular hypertrophy, and direct podocyte injury also leads to indistinguishable findings of segmental glomerulosclerosis. To comprehend how these heterogeneous injuries may lead to FSGS, it is important to understand the structure and physiologic function of the podocyte. A brief account is given below. It is clear that numerous podocyte gene products are required to construct the podocyte body and foot processes (FPs). For example, nephrin (*NPHS1*) and podocin (*NPHS2*) are the major components of the slit diaphragm (SD). CD2-associated protein (*CD2AP*) and α-actinin-4 (*ACTN4*) link the SD to the actin cytoskeleton of the FPs. Podocalyxin localized on the apical membrane and α3β1 integrin on the podocyte basolateral membrane are also required for FP integrity. Furthermore, the podocyte synthesizes the major glomerular basement membrane (GBM) components. Defective extracellular matrix synthesis by the podocyte can lead to loss of normal glomerular filtration. Mutations in structural podocyte genes cause FSGS in humans.

**Table 1:** Etiologic classification of FSGS

Primary (idiopathic) FSGS
Secondary FSGS
1. Genetic mutations
*NPHS1*
*NPHS2*
*CD2AP*
*TRPC6*
*ACTN4*
*INF2*
*ANLN*
*ARHGAP24*
*ARHGDIA*
*WT-1*
*LMX1B*
*LAMB2*
*PAX2*
*COQ2, COQ6, PDSS2, ADCK4*
2. Virus associated
HIV
Parvovirus B19
3. Medication
Heroin
Interferon-α
Lithium
Pamidronate/alendronate
Anabolic steroids
4. Adaptive structural-functional responses e.g., glomerular hypertrophy or hyperfiltration
4.1 Reduced kidney mass
Oligomeganephronia
Unilateral kidney agenesis
Kidney dysplasia
Reflux nephropathy
Surgical kidney ablation
Chronic allograft nephropathy
Any advanced kidney disease with reduction in functioning nephrons
4.2 Initially normal kidney mass
Diabetes mellitus
Hypertension
Obesity
Cyanotic congenital heart disease
Sickle cell anemia
5. Malignancy (lymphoma)
6. Nonspecific pattern of FSGS caused by kidney scarring
Focal proliferative glomerulonephritis (IgA nephropathy, lupus nephritis, pauci-immune focal necrotizing and crescentic glomerulonephritis)
Hereditary nephritis (Alport syndrome)
Membranous glomerulopathy
Thrombotic microangiopathy

Modified from reference [90]

The complex structural podocyte composition is also achieved by sophisticated metabolic and energy requirements, for example, autophagy and P53-dependent signaling [8]. Enzymes and kinases involved in the mitochondrial respiratory transport chain (*COQ2* [9], *COQ6* [10], and aarF domain containing kinase 4 *(ADCK4)* [11]) are also implicated in podocyte integrity; mutations in *COQ2* are implicated in collapsing FSGS.

Injured podocytes attempt to avoid death and regenerate. For example, mitotic catastrophe, a mechanism of podocyte death, represents dividing podocytes unable to complete the cell cycle and succeed in producing daughter podocytes [12]. A different source of potential replacement of injured podocytes under certain conditions are transformation of parietal epithelial cells to visceral podocytes [13]. Whether or not these failed attempts to repair podocyte injury may participate in the pathogenesis of FSGS remains to be further studied. Here we discuss major pathogenic mechanisms that have been well documented.

#### Genetic causes of FSGS

Human genetic studies in the past two decades have demonstrated that FSGS is primarily a podocytopathy with more than 20 mutated podocyte genes confidently implicated in the pathogenesis of NS/FSGS [14]. These mutated genes can be divided into the following categories: (a) SD-associated molecules, (b) podocyte cytoskeleton related molecules, (c) podocyte transcription factors, and (d) adhesion and extracellular matrix molecules. (a) SD-associated molecules include nephrin, podocin [15], CD2AP, and transient receptor potential cation channel 6 (*TRPC6*). Mutated *NPHS1* was the first podocyte gene identified in congenital NS (CNS) of the Finnish type [16]. This discovery revolutionized our understanding of the pathogenesis of NS/FSGS. CD2AP is a 70 KD adaptor/linker protein involved in regulation of the actin cytoskeleton and intracellular trafficking [17, 18]. CD2AP also links podocin and nephrin to the phosphoinositide 3-OH kinase [19]. TRPC6 functions as a podocyte calcium influx pathway and upstream regulator of podocyte cytoskeleton [20]. (b) Podocyte cytoskeleton related molecules include α-actinin-4 [21], inverted formin 2 (*INF2*) [22], and anillin (*ANLN*) [23]. Their mutations impair the integrity of the podocyte actin cytoskeleton [23–25]. Mutated *INF2* is the most common cause of autosomal dominant (AD) FSGS. Recently, mutations in *ARHGDIA* [26] and *ARHGAP24* [27] and increased expression of podocyte-specific *RAP1GAP* [28] were shown to regulate small GTPases including Rac1 and RAP1, thereby dysregulating the podocyte actin networks. In addition, podocyte endocytosis involving dynamin, synaptojanin, and endophilin proteins is important for the maintenance of the glomerular filtration barrier (GFB) via an action on actin dynamics [29]. (c) Mutations in podocyte transcription factors *LMX1B* and WT-1 cause Nail-patella syndrome [30, 31] or Denys-Drash/Frasier syndrome [32] respectively. Moreover, the WT1-R458Q mutation was reported recently as the cause of nonsyndromic AD FSGS [33]. (d) Mutations in adhesion and extracellular matrix molecules such as integrins and laminin-β2 (*LAMB2*) play an important role in the pathogenesis of FSGS. Mutations in *LAMB2* cause Pierson syndrome (OMIM 609049), which is characterized by CNS/diffuse mesangial sclerosis, severe ocular abnormalities, and neurodevelopmental impairments [34–36]. Laminin, type IV collagen, nidogen, and sulfated proteoglycans comprise the GBM [37], and laminins are heterotrimeric glycoproteins containing one α, one β, and one γ chain. The major laminin heterotrimer in the mature GBM is laminin α5β2γ1, or LM-521 [38]. Laminin trimerization occurs in the endoplasmic reticulum (ER) and involves association of the three chains along their laminin coiled-coil domains to form the long arm [39]. Once trimers are secreted into the extracellular space, they polymerize to form the supramolecular laminin network via interactions among the NH2-termini of the short arms (LN domains) [40, 41]. *Lamb2* null mice recapitulate Pierson syndrome [42–47]. Although *LAMB2* null mutations cause the full syndromic phenotype of Pierson syndrome, certain *LAMB2* missense mutations, including R246Q and C321R, which are located in the LN or LEa domain of LAMB2 respectively, cause CNS with mild extrarenal features [48]. Using our established cell and knockout/transgenic mouse models resembling human NS harboring the R246Q or C321R mutation respectively, we have shown that both R246Q and C321R mutations cause defective secretion of laminin-521 from podocytes to the GBM [49, 50]. Furthermore, we have demonstrated that the misfolded C321R mutant protein induces podocyte ER stress and proteinuria *in vivo* [50].

These monogenic forms of NS/FSGS also provide a window to investigate the pathogenesis of sporadic FSGS, which is much more common and complex. For example, genetic causes were identified in 32.3-52 % of children with sporadic steroid-resistant NS (SRNS) [51, 52]. The precise glomerular morphology caused by genetic mutations may depend on the age of onset, function of the responsible gene and gene products, and other factors which are not entirely understood to date [53]. A summary of genetic mutations causing FSGS is listed in Table 1.

Besides the direct disease-causing gene mutations in FSGS, the role of genetic risk variants in FSGS has also been investigated. A classic example is apolipoprotein L1 (*APOL1*) gene risk variants-associated nephropathy [54], which is a devastating spectrum of kidney diseases including focal global glomerulosclerosis (FGGS) that was historically attributed to hypertension, FSGS or the collapsing variant, sickle cell nephropathy, and severe lupus nephritis in AAs. The risk variants G1 (S342G:I384M) and G2 (del.N388/Y389) are two coding variants in the *APOL1* gene on chromosome 22q13. The mutant alleles confer protection against trypanosomal infections in AAs at the cost of an increased risk of kidney disease. Although 51 % of AAs have at least one risk allele and 13 % have two parental risk alleles, only a subset of individuals with genetic risk develops kidney disease. It is likely that the interplay between *APOL1* and several modifiable environmental factors or interactive genes such as *NPHS2*, *SDCCAG8*, and *BMP4* produces the variable spectrum of *APOL1* nephropathy [55].

#### Circulating factors of FSGS

Shalhoub first suggested the existence of a serum factor that causes FSGS in 1974 [56]. Savin *et al*. demonstrated that a serum protein with a molecular mass between 20 and 50 kD increases GFB permeability and induces post-transplantation recurrent FSGS [57]. In addition, they proposed that the FSGS factor is a cardiotrophin-like cytokine-1 (CLC-1) [58].

#### Hemodynamic adaptations leading to glomerular hypertrophy

Glomerular hypertrophy and hyperfiltration can be associated with reduced nephron mass. For example, oligomeganephronia, unilateral renal agenesis, renal dysplasia, reflux nephropathy, secondary to surgical or traumatic ablation, chronic allograft nephropathy, and other causes of nephron loss lead to FSGS. In contrast, obesity, hypertension, cholesterol atheroembolism, cyanotic congenital heart disease, and sickle cell disease lead to glomerular hypertrophy and potentially FSGS without reduced nephron mass.

#### Direct podocyte injury

Medications such as interferon-α, lithium, and pamidronate and viruses such as HIV and parvovirus B19 can induce direct podocyte dysfunction. Several of these drugs cause a collapsing type of FSGS characterized by podocyte proliferation and implosion of the capillary tuft [59].

### Is pathogenesis reflected in the histopathology of FSGS?

**Fig. 1:**
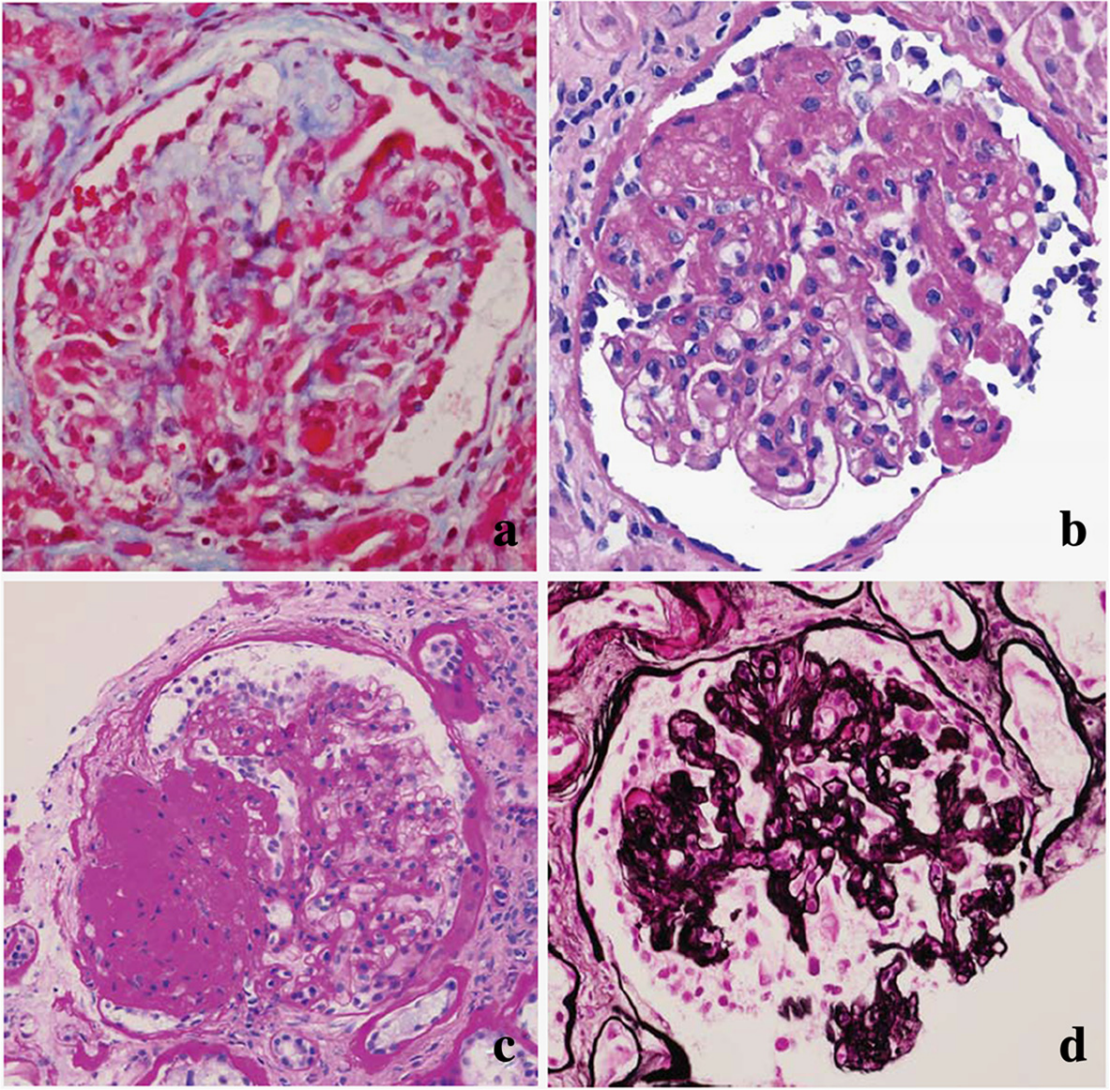
Histopathological FSGS variants. **a** Adhesion of the capillary loops to Bowman’s capsule is thought of as a nidus for segmental sclerosis and an early stage of FSGS (Trichrome). **b** FSGS with amorphous (hyaline) deposits (Periodic acid–Schiff). **c** Segmental consolidation (<50 %) of the glomerulus is typical of FSGS NOS (Periodic acid–Schiff). **d** Collapsing FSGS is characterized by segmental (or global) proliferation of podocytes and segmental (or global) implosion of the capillary loops (Jones Methenamine Silver)

FSGS is defined as segmental solidification of the glomerular capillary tuft with accumulation of extracellular matrix initiated by an adhesion between the capillary tuft and the Bowman’s capsule (synechia) (Fig. 1a). Hyalinosis (Fig. 1b) and foam cells can also be present. The scarred segment can be perihilar or at the tip of the glomerulus (tip lesion). Segmental sclerosis or hyalinosis in any part of the glomerulus is classified as FSGS, NOS (Fig. 1c). A unique presentation of FSGS is collapsing FSGS characterized by proliferation of podocytes and implosion of the capillary tuft (Fig. 1d). While many studies have shown better prognosis for the tip lesion and worse for collapsing FSGS, the true value of classifying FSGS based on morphology has been debated, particularly when it comes to collapsing FSGS which shows no segmental solidification but implosion of the capillary loops and podocyte proliferation instead. In addition, the morphologic variants of FSGS fall short in distinguishing primary from secondary forms of FSGS. A recent study has proposed that adult FSGS patients presenting with NS, extensive FP effacement (≥80 %) on electron microscopy (EM) examination, and no risk factors associated with secondary FSGS are likely to have primary FSGS. Conversely, the absence of NS in a patient with segmental FP effacement on EM strongly suggests a secondary FSGS [60]. However, distinction between primary and secondary FSGS may not be clear-cut sometimes. For example, patients with two *APOL1* renal risk alleles are prone to develop hypertension and chronic kidney disease complicated by FSGS [61]. In such patients, is FSGS primarily due to a specific genetic predisposition or secondary to hypertension-induced hyperfiltration?

Barisoni et al. proposed a taxonomy for the podocytopathies that classifies along two dimensions: histopathology, including podocyte phenotype and glomerular morphology (minimal change nephropathy (MCN), FSGS, diffuse mesangial sclerosis (DMS), and collapsing glomerulopathy (CG)), and etiology (idiopathic, genetic, and reactive forms). Three distinct pathways of injury and repair characterize the podocytopathies. First, in MCN, podocyte injury is limited to FP effacement and podocyte number remains normal. Second, a more severe form of podocyte injury may cause podocyte detachment and death, thereby initiating an injury cascade that results in the segmental scar characteristic of FSGS. Third, podocyte injury may lead to either low rates of podocyte proliferation manifesting as DMS or high rates of proliferation manifesting as CG. Whenever possible, final diagnosis of the podocytopathies should include three elements: morphologic entity, etiologic form, and specific pathogenic mechanism [62]. This proposal is supported by recent studies that show defining patients by the underlying disease mechanism improves patient management [33, 51, 52, 55].

### Genetic screening in clinical practice and proposed stratification of patients with FSGS

Sanger sequencing is expensive and results can take weeks or even months. Therefore, the following questions need to be considered before advising a genetic testing in routine clinical practice [63].

#### Does the result of genetic testing affect treatment decisions?

Most studies have indicated that genetic forms of FSGS are steroid-resistant [64, 65] and most likely will not respond to immunosuppressive therapy with alkylating agents. However, mutation analysis should not be used to discard cyclosporine (CSA) as a therapeutic agent. Recently, it has been shown that the *APOL1* risk genotype does not influence proteinuria responses to CSA or mycophenolate mofetil (MMF)/dexamethasone in idiopathic FSGS patients enrolled in the National Institutes of Health (NIH)-sponsored FSGS Clinical Trial (FSGS-CT) [66].

#### Does the result of genetic testing influence care beyond glomerular disease?

Mutations in some genes including *WT-1* [67, 68], mitochondrially encoded tRNA leucine 1 [69], *LAMB2* [70], *ITGB4* [71], *CD151* [72, 73], *SCARB* [74], *LMX1b* [31], and non-muscle myosin IIa (*MYH9*) [75] can have extra-renal manifestations. Thus, in syndromal forms of FSGS, additional studies to exclude extra-renal disease may be needed necessitating important additional management considerations for such patients.

#### Does the result of genetic testing help in family planning?

Mutation analysis should be considered in all children with CNS since mutation detection rate is almost 100 % [76]. Even though not all CNS show FSGS on renal biopsy, the majority are indeed either FSGS NOS or collapsing FSGS. Genetic testing should also be performed in children with familial and sporadic SRNS; the prevalence of genetic causes of SRNS could be as high as 52 % [51]. In addition, genetic screening should be considered in adults with a family history of FSGS. Genetic screening is of limited value in adult patients with sporadic FSGS, with the exception of screening for the podocin p. R229Q in young adults since compound heterozygosity for p.R229Q coupled with a pathogenic *NPHS2* mutation is associated with adult-onset SRNS, mostly among patients of European and South American origin. Screening for the p.R229Q variant is recommended in these patients, along with further *NPHS2* mutation analysis in those carrying the p.R229Q variant [77].

#### Does the result of genetic testing impact decisions related to kidney transplantation?

In SRNS/FSGS, the detection of a homozygous or compound heterozygous mutation will predict a low risk of recurrence post transplantation. This knowledge should be reassuring for patients and their parents. However, mutated nephrin (*NPHS1*) is an exception to the rule. Recurrence rate post transplantation was 37 % in CNS patients with the genotype of Fin-major/Fin-major, which is a 2-base pair deletion in exon 2 of *NPHS1*, but not in any other genotypes. The development of high levels of circulating anti-nephrin antibodies likely contributes to FSGS recurrence [78].

To determine whether *APOL1* genotyping should be performed broadly in deceased kidney donors with African ancestry, *APOL1* G1 and G2 variants were genotyped in newly accrued DNA samples from AA deceased donors of kidneys recovered and/or transplanted in Alabama and North Carolina in a recent study. *APOL1* genotypes and allograft outcomes in subsequent transplants from 55 U.S. centers were analyzed. For all 675 kidneys transplanted from donors at both centers, kidneys from AA deceased donors with two *APOL1* nephropathy variants reproducibly associate with higher risk for allograft failure after transplantation (HR 2.26; p = 0.001) [79]. The new study validates a prior single-center report [80]. These findings warrant consideration of rapidly genotyping deceased AA kidney donors for *APOL1* risk variants at organ recovery.

#### What are the possible implications of whole genome (exome) sequencing?

Next generation sequencing (NGS) is rapidly transforming the genetic testing of FSGS [81]. It is likely that whole exome screening will be available for the clinical diagnostic use in the next few years at much lower costs. The high throughput DNA sequencing technology will enable us to analyze multiple NS-causing podocyte genes in one array, to clarify genotype-phenotype relationships, and to explore the role of genetic epistasis (combinations of genetic heterozygosity in different recessive genes) in the pathogenesis of FSGS. Moreover, the advent of NGS has led to a rapid discovery of novel genetic variants in known or novel FSGS-causing genes. In a recent study, one patient with presumed secondary FSGS due to congenital vesicoureteral reflux was surprisingly revealed to have two deleterious *COL4A3* mutations associated with Alport syndrome (AS) and a concurrent novel deleterious *SALL2* mutation linked to renal malformations [82]. Likewise, in a cohort of 70 families with a diagnosis of hereditary FSGS, 10 % of cases were identified to carry rare or novel variants in *COL4A3* or *COL4A4* known to cause AS [83]. *PAX2* mutations, which have been shown to lead to congenital abnormalities of the kidney and urinary tract, may also contribute to adult-onset AD FSGS in the absence of overt extrarenal manifestations [84]. Thus, targeted or whole exome sequencing integrated with clinicopathological information can reveal novel and rare gene mutations and provide insights into etiologies of complex renal phenotypes with equivocal clinical and pathologic presentations [82]. A major challenge ahead in NGS is to determine the actual pathogenicity of large amounts of identified missense variants due to lack of mechanism-based, high-throughput functional assays.

### Treatment of FSGS

#### Treatment of secondary FSGS

Attempts to treat the primary etiology of FSGS should be the initial step. For example, FSGS secondary to obesity and heroin remits after weight reduction or cessation of heroin use [85]. Highly active antiretroviral therapy (HAART) has been proven useful for HIV-associated nephropathy [86]. There is no evidence to suggest corticosteroids or immunosuppressive therapy in the treatment of secondary FSGS.

#### Treatment of idiopathic FSGS in adults

The potential efficacy of therapy must be considered in relation to the natural history of the disease. The rate of spontaneous remission among patients with NS is unknown. A study reported that after a median follow-up of 9.4 years, 13 out of 20 idiopathic FSGS patients with nephrotic-range proteinuria and normal renal function achieved spontaneous complete or partial remissions of proteinuria (65 %). However, due to the small number of patients in this study, we cannot draw a definite conclusion [87]. Most studies showed that untreated primary FSGS often followed a progressive course to ESRD [88, 89].

For the initial treatment of FSGS, the Kidney Disease Improving Global Outcomes (KDIGO) 2012 guideline [90] recommended that corticosteroid and immunosuppressive therapy be considered only in idiopathic FSGS associated with clinical features of the NS (1C). KDIGO suggested prednisone be given at a daily single dose of 1 mg/kg (maximum 80 mg) or alternate-day dose of 2 mg/kg (maximum 120 mg) (2C). It also suggested that the initial high dose of corticosteroids be given for a minimum of 4 weeks up to a maximum of 16 weeks, as tolerated, or until complete remission has been achieved, whichever is earlier (2D). Calcineurin inhibitors (CNIs) are considered first-line therapy for patients with relative contraindications or intolerance to high-dose corticosteroids (e.g., uncontrolled diabetes, psychiatric conditions, severe osteoporosis) (2D). (Based on the KDIGO 2012 guideline, the strength of recommendation was indicated as level 1 or level 2, and the quality of the supporting evidence was shown as A, B, C, or D. Level 1: “we recommend”; Level 2: “we suggest”. The quality of evidence was stratified into different grades: A-high, B-moderate, C-low, and D-very low [91]). A variety of nonrandomized retrospective studies have reported that prednisone induces 40 to 80 % rates of complete or partial remission.

#### Treatment of SR FSGS

For SR FSGS, the KDIGO 2012 guideline suggested that CSA at 3–5 mg/kg/d in divided doses be given for at least 4–6 months (2B). If there is a partial or complete remission, continue CSA treatment for at least 12 months, followed by a slow taper (2D). The guideline also suggested that patients, who do not tolerate CSA, be treated with a combination of MMF and high-dose dexamethasone (2C) [90].

The North American Nephrotic Syndrome Study Group including 12 clinical centers in North America conducted a well-designed clinical trial of CSA in SR FSGS patients [92]. In this study, all patients previously failed to achieve a remission of the proteinuria after a minimum of eight weeks of prednisone at ≥ 1 mg/kg/day. The major entry criteria were proteinuria ≥ 3.5 g/d and creatinine clearance ≥ 42 ml/min/1.73 m^2^. Patients with CG were excluded. 26 weeks of CSA treatment plus low-dose prednisone was compared to placebo plus prednisone. Despite relapses after CSA was discontinued, at the end of long term follow-up of 104 weeks, there were still significantly more remitters in the CSA-treatment group. In addition, it has been found that CSA can directly stabilize podocyte actin cytoskeleton [93]. There are no randomized clinical trials using tacrolimus. Uncontrolled studies suggest that tacrolimus may be an alternative in patients intolerant of CSA [94, 95].

In a recent NIH-funded multicenter randomized FSGS Clinical Trial (FSGS-CT), the efficacy of a 12-month course of CSA was compared to a combination of MMF and oral pulse dexamethasone (DEX) in children and young adults with SR primary FSGS [96]. In the CSA arm, CSA was given at 5–6 mg/kg/day for 12 months with a targeted 12 h trough level of 100–250 ng/ml. In the MMF + DEX arm, 25–36 mg/kg/day of MMF were given in addition to 46 pulse doses of DEX for 12 months. In addition, both arms were treated with prednisone, 0.3 mg/kg, every other day for the first 6 months and angiotensin-converting enzyme inhibitor (or angiotensin receptor blocker) for 18 months. The primary outcome was based on achievement of partial and complete remission during the first 52 weeks. The main secondary outcome was sustainable remission in proteinuria after withdrawal of immunosuppressive agents during weeks 52–78. There was no statistical difference in the primary outcome or the main secondary outcome between the two therapies. However, there are important limitations in this study that have hindered drawing firm conclusions [97]. Other smaller observational studies have suggested a possible benefit of MMF given with or without steroids [98–101].

#### Alternative & Novel therapies for FSGS

Table 2 lists novel therapies based on different disease mechanisms and most of them are still under clinical investigation. It is worthwhile pointing out that plasmapheresis is successful in treating some patients with post-transplantation recurrent FSGS [57]. However, it has not been proven to be useful in patients with FSGS in their native kidneys. Rituximab is a genetically engineered chimeric murine/human monoclonal IgG1 antibody directed against the CD20 antigen expressed in human B cells. There are conflicting results regarding the use of rituximab in FSGS, and it has been unclear exactly how this drug achieves success in some patients, but not others [102, 103].

**Table 2:** Alternative/novel treatments for FSGS

Circulating factors
• Plasmapheresis/Immunoabsorption [57]
• Galactose [106]
Immune modulation
• Rituximab
• Adrenocorticotropic hormone (ACTH) [107–109]
Anti-fibrotic therapy
• Tumor necrosis factor (TNF): Adalimumab, a human anti-TNF monoclonal antibody
• Connective tissue growth factor (CTGF) [110]: FG-3019, a human monoclonal antibody against CTGF
• Transforming growth factor β (TGF-β) [110]: Fresolimumab, a human monoclonal antibody directed against human TGF-β1, 2 and 3
• Pirfenidone [111, 112]: multifaceted roles

In the era of personalized medicine, identifying FSGS-causing gene mutations and investigating their underlying molecular mechanisms have immense potential for the development of highly-targeted therapy. For example, CoQ_10_ supplementation can attenuate proteinuria in SRNS patients carrying mutations in CoQ10 biosynthesis pathway genes like *COQ2, COQ6*, and *ADCK4* [10, 11, 104].

Additionally, other novel therapies suggested from mouse studies have not yet been tried in humans. For example, retinoid acid exerts important anti-proteinuric, anti-fibrotic, and anti-inflammatory effects in multiple experimental models of kidney disease, possibly through promoting renal progenitors differentiation and podocyte regeneration [105].

## Conclusions

FSGS is the leading cause of ESRD due to primary glomerular disease in the U.S. and is increasing in incidence. Seminal human genetic studies have illuminated podocyte dysfunction as the major contributor to GFB failure in this disease. Mutations in >20 podocyte genes have been implicated as causal factors for Mendelian forms of FSGS. Meanwhile, the understanding of *APOL1* genetic risk variants in conferring susceptibility to common kidney diseases, including FSGS, chronic kidney disease, and hypertension, is evolving. In addition, the development of NGS has revealed that FSGS can arise from mutated genes previously only implicated in AS and congenital urogenital anomalies (for example*, COL4A3*, *COL4A4*, *PAX2 or SALL2*) and will further accelerate the discovery of novel podocyte genes or genetic variants linked to FSGS. The technological breakthroughs will transform risk assessment, the diagnostic pathologic schemes currently used, and treatment of FSGS. More than ever before, there is need for understanding the underlying molecular mechanisms, evaluating genotype-phenotype correlations, and design of clinical trials in a highly-targeted manner.
